# The Atrioventricular Conduction Axis Revisited for the 21st Century

**DOI:** 10.3390/jcdd10110471

**Published:** 2023-11-19

**Authors:** Damian Sanchez-Quintana, Andrew C. Cook, Yolanda Macias, Diane E. Spicer, Robert H. Anderson

**Affiliations:** 1 Department of Human Anatomy and Cell Biology, Faculty of Medicine, University of Extremadura, 06006 Badajoz, Spain; 2 Institute of Cardiovascular Science, University College London, London WC1E 6BT, UK; a.cook@ucl.ac.uk; 3 Department of Medical and Surgical Therapeutics, Faculty of Veterinary, University of Extremadura, 10071 Cáceres, Spain; yolandamg@unex.es; 4 Heart Institute, Johns Hopkins All Children’s Hospital, St. Petersburg, FL 33701, USA; spicerpath@hotmail.com; 5 Biosciences Institute, Newcastle University, Newcastle upon Tyne NE1 7RU, UK; sejjran@ucl.ac.uk

**Keywords:** atrioventricular node, bundle of His, bundle branches, Mahaim conduction

## Abstract

Although first described in the final decade of the 19th century, the axis responsible for atrioventricular conduction has long been the source of multiple controversies. Some of these continue to reverberate. When first described by His, for example, many doubted the existence of the bundle we now name in his honour, while Kent suggested that multiple pathways crossed the atrioventricular junctions in the normal heart. It was Tawara who clarified the situation, although many of his key definitions have not universally been accepted. In key studies in the third decade of the 20th century, Mahaim then suggested the presence of ubiquitous connections that provided “paraspecific” pathways for atrioventricular conduction. In this review, we show the validity of these original investigations, based on our own experience with a large number of datasets from human hearts prepared by serial histological sectioning. Using our own reconstructions, we show how the atrioventricular conduction axis can be placed back within the heart. We emphasise that newly emerging techniques will be key in providing the resolution to map cellular detail to the gross evidence provided by the serial sections.

## 1. Introduction

Over their history of 130 years, the substrates for atrioventricular conduction have spawned multiple controversies. Towards the end of the 19th century, the experiments of Stannius, coupled with the investigations of Gaskell [1], had established that conduction was myogenic rather than neurogenic. The investigations at that time, however, had been conducted on frogs and turtles. There was no information regarding the pathways for atrioventricular conduction in the human heart. Controversy began in the last decade of the century, when His described a solitary bundle that crossed the atrioventricular junctions [2]. Kent, in contrast, publishing in the same year, maintained he had discovered multiple pathways in normal mammalian hearts [3]. It is surprising that others working in the field where unable immediately to resolve this initial disagreement. As consummate an anatomist as Keith, for example, in the first decade of the 20th century, had expressed scepticism regarding the very existence of the bundle described by His [4]. Such matters at that stage were largely debates between anatomists. The discovery of the ability to deduce cardiac actions through the electrocardiograph brought these issues into the clinical arena. It was through his interactions with the pioneering cardiologist Mackenzie that Keith was able subsequently to banish his uncertainties regarding the presence and location of the atrioventricular bundle [5]. Mackenzie had provided Keith with the monograph written by Tawara, a Japanese working under the direction of Aschoff [6]. As Keith commented in his autobiography [7], with the discovery of the atrioventricular conduction axis by Tawara, heart research entered a new epoch. 

Despite this recognition, the conduction axis suffered significant vicissitudes in the middle of the 20th century, not least with suggestions made that it did not exist [8]. The emergence of cardiac surgery gave the lie to that notion, as surgeons recognised that improperly placed sutures could produce atrioventricular dissociation. The studies of Lev [9] and Truex [10] served to steady the ship. If we are to judge by a drawing recently produced to show the location of the bundle as a guide to pacing [11], nonetheless, clinicians still have much to learn if they are to appreciate the precise location of the components of the axis relative to gross anatomical landmarks. Were the left bundle branch truly located as shown in the drawing [11], then there would be no reason to fear the induction of the problems with ventricular conduction that continue to plague transcatheter insertion of aortic valves [12]. There remains a need, therefore, to provide a description of the arrangement of the atrioventricular conduction axis as it lies within the heart that is fit for the 21st century. We hope that our current review provides the basis for such descriptions.

## 2. Historical Disagreements

**Figure 1:**
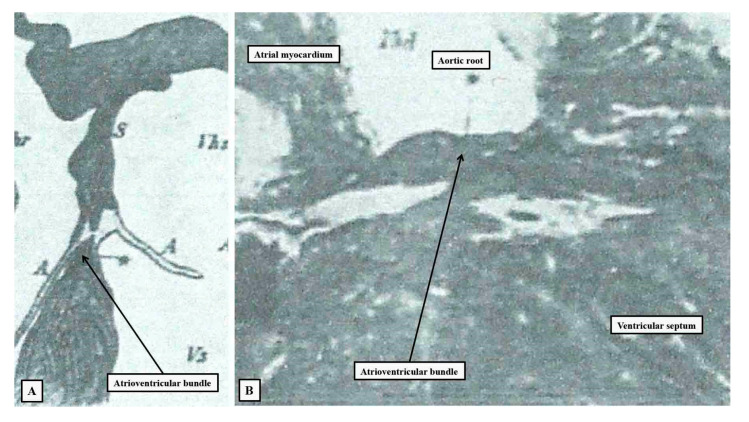
The illustrations have been taken from the initial publication by Wilhelm His Junior [2], and re-labelled. Panel (**A**) shows the short axis of the non-branching atrioventricular bundle, with panel (**B**) showing the course of the bundle relative to the aortic root as seen from the right side.

As we have already indicated, in 1893, two investigators offered markedly different accounts of the alleged substrates for atrioventricular conduction. Wilhem His Jr was working in the department of his father, who was then perhaps the foremost anatomist and embryologist practising in Europe, having invented the microtome and thereby the process of “serial sectioning” followed by three-dimensional reconstruction. His Junior described the bundle as attaching “itself along the upper margin of the ventricular septal muscle by means of numerous fiber exchanges; proceeding on top of this toward the front until near the aorta it forks itself into a right and left limb”. He illustrated the arrangement in both the short and long axis of the bundle (Figure 1). 

**Figure 2:**
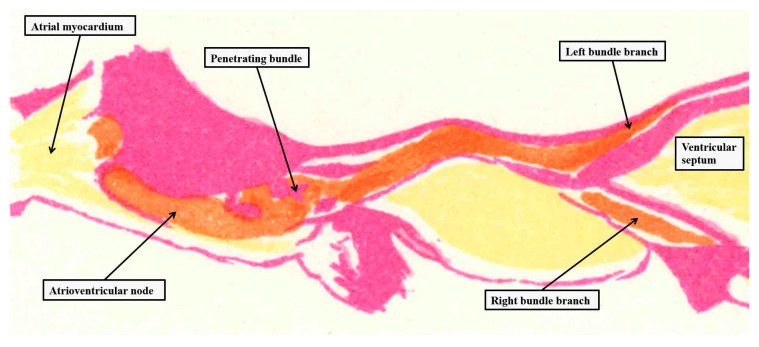
The image is taken from one of the plates prepared by Tawara to show the overall arrangement of the atrioventricular conduction axis [6] and reorientated to approximate to an attitudinally appropriate arrangement, with the atrial chambers seen to the left hand, the ventricular chambers to the right hand, and the left-sided chambers to the top of the panel. The purple shading shows the insulating tissues of the atrioventricular junctions, with yellow showing working myocardium and orange the conduction axis.

It is surprising, based on these images, that Keith was unable to find the structure during his initial investigations [4]. As we will explain, however, Keith was initially depending on gross dissection to find the bundle. The added value offered by Tawara was the finding, using analysis of serial histological sections, that the axis commenced in the atrioventricular node (Figure 2) and terminated as the ramifications of the ventricular bundle branches. 

**Figure 3:**
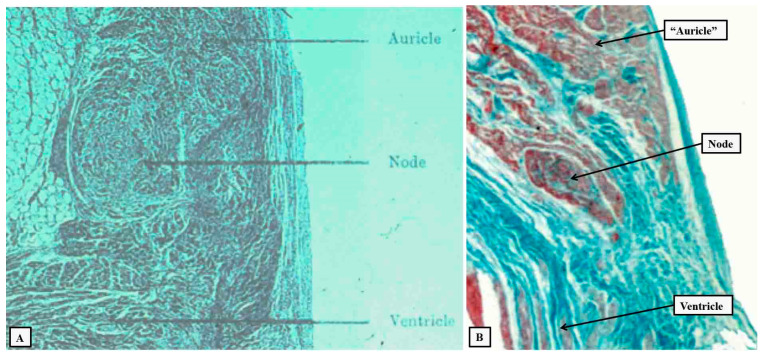
Panel (**A**) is a reproduction of the image presented to the Physiological Society by Kent in 1913 [15], allegedly to demonstrate the presence of a lateral right-sided atrioventricular pathway. Kent labelled the structure as a “node”. As is shown in panel (**B**), such entities do exist, but in the normal heart they are sequestered within the vestibule of the tricuspid valve.

Tawara’s investigations, however, had not been straightforward. It was not until he had studied the ovine heart that he was able to trace the bundle branches into their continuity as the cardiomyocytes described much earlier by Purkinje. Overall, Tawara spent nearly three years studying in Marburg under the direction of Ludwig Aschoff. On the basis of letters included in the English translation of his monograph [13], we know that, in describing his initial research, he commented “when I then considered the failure of my experiments, I was deeply unhappy. During these two long years I did not have one single happy day”. It was only in the final months of his stay in Marburg that he put together the monumental descriptions that served to clarify the seeming conflict between the descriptions offered by His on the one hand [2] and Kent on the other [3]. In comparison to the description provided by His for the myocardial connection he had discovered (Figure 1), the writings of Kent are, at best, vague. His illustrations, furthermore, are less than convincing. Despite this, when recounting the history of his own discovery, His was prepared to give Kent the benefit of the doubt. As translated by Bast and Gardner [14], His, when summarising the situation, commented that “the anatomical description of the conduction bundle is not very clear, but one can assume from his Figure 3 of Plate 12 that he saw that part of the A-V bundle which passes from the auricle towards the septum ventriculorum”. Having assessed the figure ourselves, we would suggest that His was generous in making this assumption. Our interpretations are in keeping with the opinions of Keith and Flack. When they reviewed the writings of Kent [4], they stated “the muscular connexion, he said, was of two kinds: 1. By direct continuity of the auricular and ventricular musculature at certain points; one of the points he specified was at the junction of the interauricular and interventricular septa of the heart; it is this point of muscular continuity which is now spoken of as the auriculo-ventricular bundle of His. 2. He described an intermediate continuity by means of a network of primitive fusiform muscular fibres which are imbedded in the fibrous tissue of the auriculo-ventricular rings of the heart. The second or intermediate muscular union described by Kent we have failed to find in the human heart”.

It was the second type of connection that Kent continued to maintain was responsible for atrioventricular conduction in the normal heart. He had made a presentation to the Physiological Society in 1913 in which he claimed to have demonstrated a lateral connection in the right atrioventricular junction (Figure 3A; [15]). The structures that he illustrated do exist. In the normal heart, however, they are sequestered within the vestibule of the tricuspid valve (Figure 3B).

We now know that they are remnants of the ring of specialised myocardium that, in the developing heart, surrounds the primary interventricular communication [16]. For many years, those interpreting the electrocardiographic features of Wolff–Parkinson–White syndrome and accepting the findings of Kent at face value considered the accessory muscular atrioventricular pathways that are part of the re-entry circuit to be “bundles of Kent” [17]. Öhnell had shown, however, that the substrate for the commonest form of the syndrome was made of working myocardium [18]. On occasion, the nodal remnants can, indeed, be part of an accessory atrioventricular pathway [19]. These pathways are now usually described as atrio-fascicular tracts. They are recognised as producing so-called “Mahaim” pre-excitation [19]. Kent, nonetheless, continued to believe that the lateral pathways were part of the normal mechanism for atrioventricular conduction, promulgating this notion as late as 1930 in an anonymous fashion when recording the proceedings of the British Association for Science [20].

**Figure 4:**
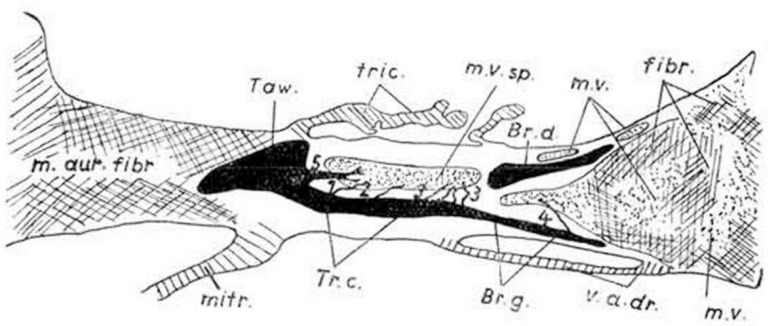
The drawing is taken from the book published by Mahaim in 1931 [21]. It shows the “superior septal pathways”, numbered 1 through 5, which Mahaim suggested provided a “paraspecific” system for atrioventricular conduction [24].

At around the same time, Mahaim had himself conducted an extensive investigation of the atrioventricular conduction axis, correlating the morphological findings with his electrocardiographic recordings. He initially published his results in a book, writing in the French language [21]. With his colleagues, he subsequently produced a series of much shorter accounts [22,23,24]. Mahaim had discovered that, although the atrioventricular conduction axis was the solitary myocardial pathway crossing the insulating plane of the atrioventricular junctions in the normal heart, the pathway was not insulated in its entirety from the underlying ventricular myocardium as it extended along the crest of the muscular ventricular septum. Describing the alternative pathways as “superior septal connections”, he illustrated them as passing directly to the ventricular myocardium from the node, the non-branching, and the branching parts of the conduction axis (Figure 4). 

Ignored for many years other than as one of the potential pathways for pre-excitation, we now know that such fasciculo-ventricular connections are to be found in the majority of normal hearts [25]. Nodoventricular connections, which can be found with some frequency in fetal hearts, have largely been obliterated by the time of birth [26].

## 3. The Atrioventricular Conduction Axis

**Figure 5:**
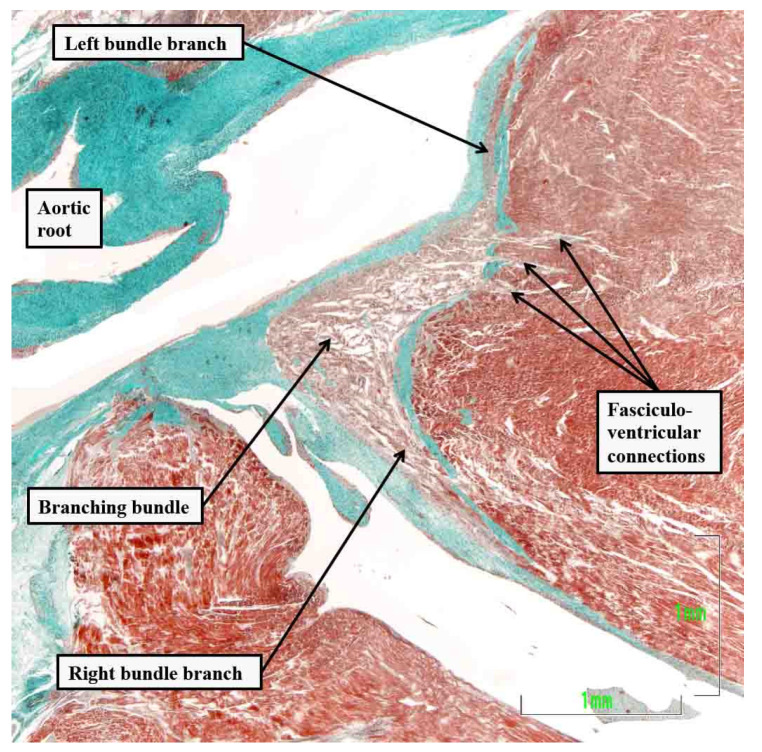
The section is taken from a fetal heart at 30 weeks’ gestation, with the section cut in a sagittal fashion across the crest of the muscular ventricular septum. It shows multiple fasciculo-ventricular connections extending between the branching atrioventricular bundle and the septal working myocardium. These pathways are to be found in the majority of hearts subsequent to birth [25].

It is now accepted that the pathway as initially observed by His, and shown by Tawara to originate in the atrioventricular node, is the solitary myocardial connection, in the normal heart, existing between the atrial and ventricular myocardial masses. And, as was shown by Mahaim, it has now been confirmed [25] that multiple short myocardial pathways extend from the axis to join the crest of the ventricular septum (Figure 5).

**Figure 6:**
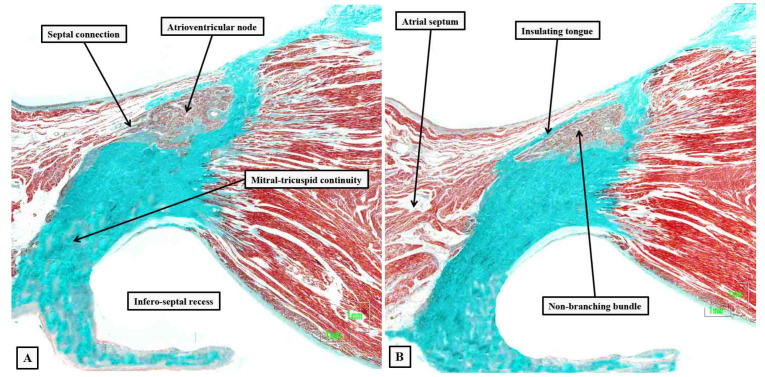
Serial histological sections, cut through the apex of the triangle of Koch normal to the hinge of the septal leaflet of the tricuspid valve of an adult human heart, which is shown at the top of the panels, showing the criterion proposed by Tawara to distinguish between the compact atrioventricular node (panel **A**) and the non-branching atrioventricular bundle (panel **B**). The distinguishing feature is the tongue of fibrous tissue that, in panel (**B**), separates the axis from the working atrial myocardium.

These superior connections were described by Mahaim as being “paraspecific” [24]. He had envisaged that they might provide an alternative system for ventricular activation in the setting of bundle branch block. It is possible that could prove to be the case, albeit conclusive evidence of their functionality remains to be established. Apart from the paraspecific connections, nonetheless, the axis itself, having been insulated from the atrial myocardium, continues as the right and left bundle branches, which extend to ramify within the apical ventricular components. It was such insulation from the adjacent working myocardium that was emphasised as a feature of conducting tracts by Aschoff and Monkeberg. They provided their definitions when seeking to defuse the debate that had emerged at the end of the first decade of the 20th century regarding the presence of a “specialised” pathway for internodal atrial conduction. Thorel had suggested that such a pathway was to be found running between the sinus and atrioventricular nodes [27]. Aschoff and Monkeberg argued that the axis as described by Tawara was an exemplar of a conducting tract [28,29]. They suggested that those arguing for the presence of such tracts within the atrial walls should show that the structures in question were histologically discrete, could be traced through serial histological sections, and most importantly should be insulated from the adjacent working myocardium. In this regard, the atrioventricular node itself, although histologically discrete and traceable from section to section, does not satisfy these criteria as a conducting tract. It would be unable to fulfil its major function, namely to delay the cardiac impulse, if its cells were insulated from the working atrial myocardium. Tawara had also emphasised the point of insulation of the conduction axis from the atrial myocardium as the criterion for distinction between the atrioventricular node and the ventricular components of the conducting pathway (Figure 6).

This definition is also important, since the histological make-up of the axis does not change at the site of insulation. The presence of insulating tissues, nonetheless, separating the conducting pathway from the adjacent atrial myocardium, means that the axis is no longer able to respond to atrial events. 

**Figure 7:**
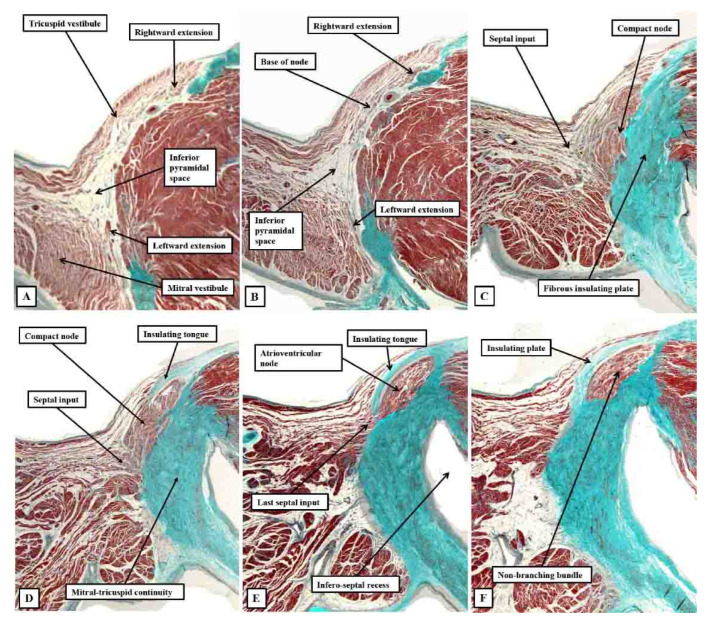
Images of serial histological sections from an adult human heart show how the components of the atrial component of the conduction axis come together in the pyramid of Koch to form the compact node, which then becomes insulated as it penetrates the fibrous tissues of the atrioventricular junctions. Panel (**A**) shows the inferior extensions in the walls of the inferior pyramidal space. Panels (**B**,**C**) show how the extensions merge to form the body of the compact atrioventricular node. The node then receives additional septal connections, as shown in panels (**D**,**E**), with the last connection, as seen in panel (**E**), representing the fast pathway into the node. Panel (**F**) then shows how the axis becomes insulated as it forms the non-branching atrioventricular bundle. It is the inferior nodal extensions, as seen in panels (**A**,**B**), which constitute the slow pathway.

The atrial component of the axis comprises the atrioventricular node and its connections with the working atrial cardiomyocytes [30]. The arrangement was well described by Tawara [6]. Using the criterions of Aschoff and Monkeberg [28,29], histologically specialised areas can be recognised within the vestibules of the tricuspid and mitral valves, areas that form the atrial walls of the pyramid of Koch (Figure 7A-Reference [31]). When traced superiorly, these specialised areas expand to become the compact atrioventricular node, which then receives direct connections from the working myocardium of the atrial septum (Figure 7B–D). The inferior extensions are now recognised as the slow pathways for nodal conduction [30]. It is the last connection between the myocardium of the atrial septum and the node that is the fast pathway (Figure 7E). Having been insulated by the fibrous tissue of the atrioventricular junction (Figure 7F), the axis then becomes the non-branching atrioventricular bundle.

**Figure 8:**
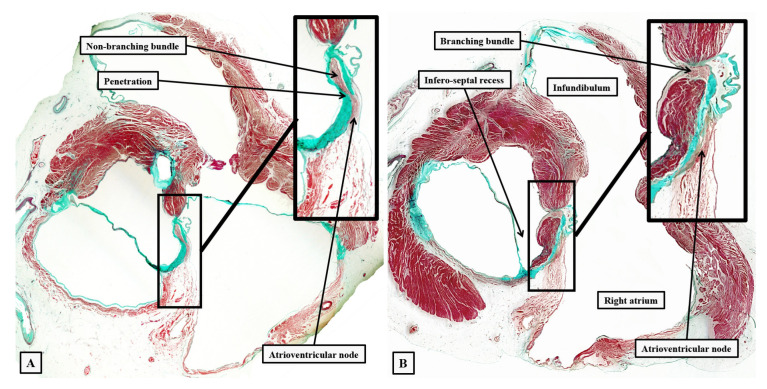
The images show short axis sections through an adult human heart cut in the plane of the atrioventricular junctions, with panel (**A**) superior to panel (**B**). The insets are magnified to show the location of the atrioventricular conduction axis. Panel (**A**) shows the atrioventricular node separated from the non-branching bundle by the insulating tissues of the atrioventricular junctions, with Panel (**B**) showing the axis branching on the crest of the muscular ventricular septum.

In their excellent review of the components of the specialised atrioventricular junction, Hecht and his colleagues suggested that the ventricular components of the axis could be divided into penetrating, non-branching, and branching components [32]. So-called “penetration”, however, is no more than the site of insulation of the axis from the atrial myocardium. We suggest, therefore, that it is sufficient simply to describe the axis as having non-branching and branching components (Figure 8). 

**Figure 9:**
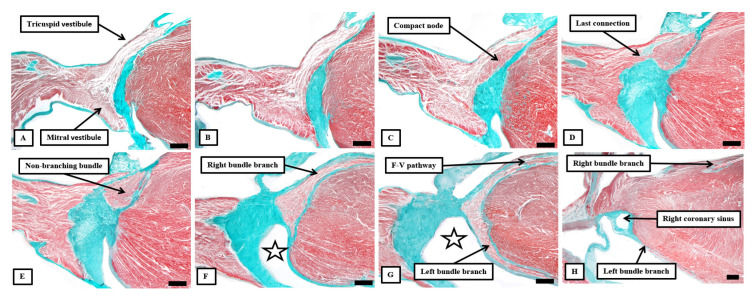
Serial sections showing the entirety of the conduction axis in a 6-month-old infant, with the plane of section comparable to that shown in Figure 7. Panels (**A**–**C**) show the formation of the atrioventricular node, with panel (**D**) showing the last septal connection. Panels (**E**,**F**) show the components of the ventricular part of the axis, with panel (**H**) showing the close approximation of the superior fascicle of the left bundle to the nadir of the right coronary aortic sinus. A fasciculo-ventricular (F-V) pathway from the right bundle branch is seen in panel (**G**). The black bar shows 1 mm. The star shows the infero-septal recess.

Having been insulated from the atrial myocardium, these ventricular parts of the axis are located on the crest of the muscular ventricular septum (Figure 9E). 

They occupy the rightward wall of the infero-septal recess of the left ventricular outflow tract (Figure 8B). Having extended superiorly for various distances, the non-branching component gives rise to the left and right bundle branches, which straddle the crest of the muscular ventricular septum (Figure 8A and Figure 9G). There is variation in the precise location relative to the septal crest, with deviation found on occasion to both the right and left sides [33]. There is currently, however, significant misunderstanding regarding the precise relationship of these ventricular components of the axis to the aortic root [11]. As shown in Figure 8 and Figure 9, the non-branching component of the axis is within the confines of the infero-septal recess, and some distance from the virtual basal plane of the aortic root. This is of obvious significance to the problem of vulnerability of the conduction axis during transcatheter replacement of the aortic valve [34,35].

## 4. The Vulnerability of the Conduction Axis 

In his original account, Tawara clearly illustrated the adjacency of the superior fascicle of the left bundle branch to the nadir of the semilunar hinges of the right coronary leaflet of the aortic root [6]. The manner of branching of the left bundle branch became a matter of controversy during the latter part of the 20th century, with Rosenbaum promoting the concept of a bifascicular left bundle so as to explain the “hemiblocks” [36]. The pupils of Rosenbaum, even now, continue to promote this concept [37,38]. It is true that bifascicular left bundles are to be found in some species, such as the ox [6,39]. Even in the ox, nonetheless, there are septal branches joining the two major fascicles together [39]. The trifascicular arrangement of the left branch, as illustrated so clearly by Tawara [6], was subsequently confirmed by Demoulin and Kulbertus [40] and by Massing and James [33]. Our own findings endorse all these observations [35]. Massing and James also emphasised that, rather than “bifurcating” to give rise to the right and left bundles, the axis gave rise to the fascicles on either side of the septum, albeit with some degree of variability [33]. They emphasised that the axis itself did not terminate having given rise to the major fascicles. Rather, it continued on the crest of the muscular ventricular septum within the aortic root as a so-called “dead-end tract” [41]. We now know that the tract is part of the ring of specialised tissue that surrounds the embryonic interventricular communication [16]. The tract cannot be identified in all hearts, but does remain as an insulated entity in some [34,41]. It should not be confused with the superior fascicle of the left bundle, as was the case in a recent description, with an accompanying commentary also failing to note the mistake [42,43]. 

**Figure 10:**
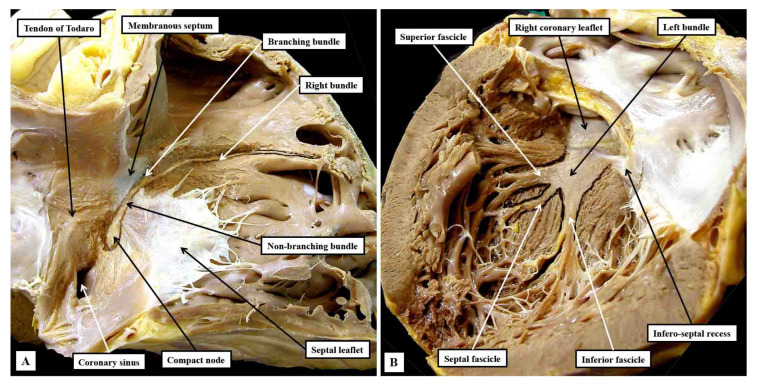
Gross dissections made to show the approximate locations of the components of the conduction axis seen relative to the right-sided (panel **A**) and left-sided (panel **B**) chambers. The ramifications of the bundle branches have been enhanced by Indian ink on the septal surfaces.

Part of the problem underscoring the distinction of the fascicle from the dead-end tract might well relate to the fact that, most frequently, clinicians do not describe the fascicles of the left bundle using attitudinally appropriate terms [44]. Rather than being located “anteriorly” and “posteriorly”, two of the fascicles of the left bundle are found superiorly and inferiorly (Figure 10B), continuing from the septum towards the supero-lateral and infero-septal papillary muscles of the mitral valve. The overall conduction axis, furthermore, extends from an inferior atrioventricular node to the superiorly located bundle branches (Figure 10A). 

**Figure 11:**
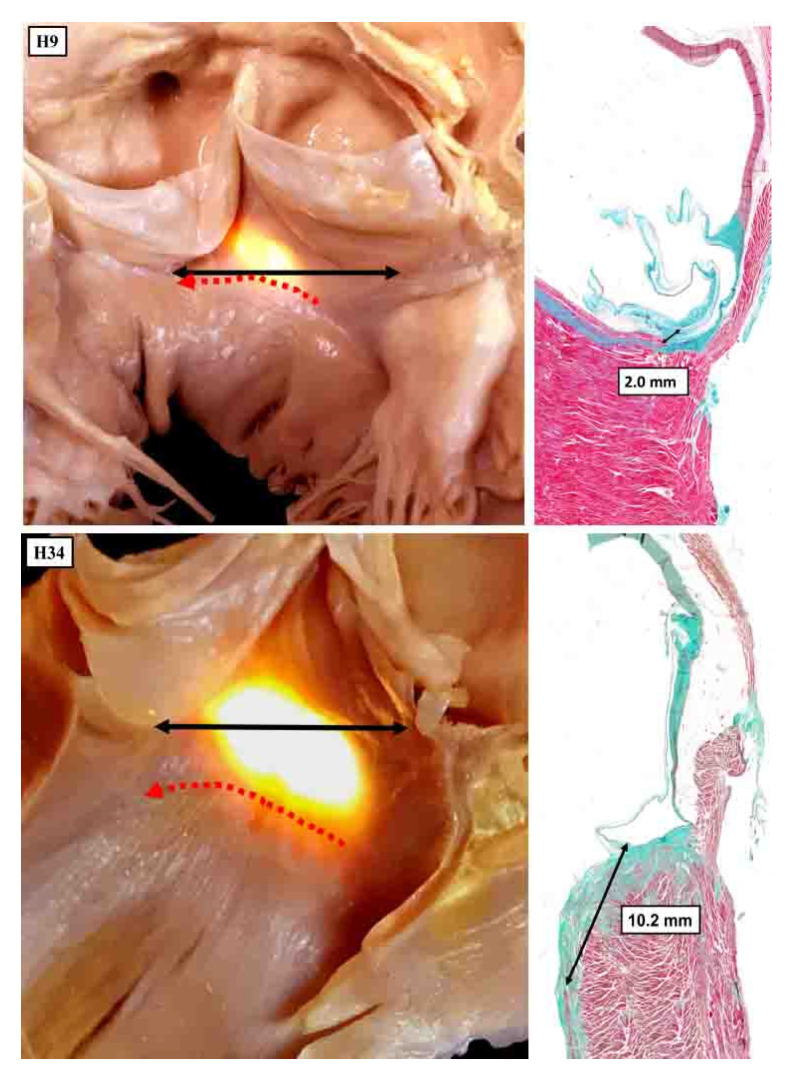
The panels show the variability in the adjacency of the superior fascicle of the left bundle to the nadir of the hinge of the right coronary aortic leaflet. In both hearts, the membranous septum has been illuminated from the right side, with the images showing the subaortic outflow tract as seen from the front. The double-headed black arrow shows the virtual basal plane of the aortic root.

It is this relationship that accounts for the relatively distant location of the node and the non-branching bundle relative to the virtual basal plane of the aortic root. The part of the axis that is closest to the aortic root, as illustrated by Tawara, is the superior fascicle of the left bundle. This can reach within millimetres of the nadir of the hinge of the right coronary leaflet (Figure 11), although there is variation in this distance [34]. 

When seen from the right side, it is difficult to be sure of the precise location of the branching bundle and the fascicles of the left bundle (Figure 10A). It is likely this difficulty that explains the bizarre situation shown in some reviews produced for those performing pacing of the axis [11]. The currently accepted landmarks for locating the axis from the right side are well established as the apex of the triangle of Koch and the medial papillary muscle of the tricuspid valve [45]. Tawara also illustrated well the course of the right bundle as a narrow cord extending from the medial papillary muscles to the moderator band of the right ventricle (Figure 10A). It is knowledge of these gross anatomical landmarks, therefore, that permits the conduction axis to be placed back within the heart.

## 5. The Gross Anatomical Landmarks

**Figure 12:**
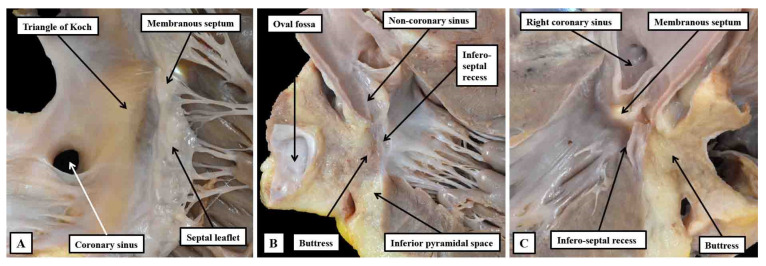
Dissections made in an infant heart to show the inter-relations between the inferior pyramidal space and the infero-septal recess of the left ventricular outflow tract. Panel (**A**) shows the right atrial wall and the adjacent septal myocardium of the right ventricle, which was removed by a section parallel to the endocardial surfaces to leave the components shown in panel (**B**). The cut passes across the ventricular septum into the left ventricular outflow tract. Panel (**C**) then shows the left-sided surface of the parts removed to produce the image shown in panel (**A**). Taken together, the cuts show how the apex of the inferior pyramidal space is directly adjacent to the inferior extent of the infero-septal recess.

With the eye of faith, and with careful dissection, it is possible for the gross anatomist to reveal the location of the components of the atrioventricular conduction axis (Figure 10). Gross dissection, however, is fraught with problems. It was because, in his initial investigations, Keith had relied on gross dissection to reveal the presence of the non-branching bundle that he doubted its existence [4]. Only when reinforcing his studies with histological investigations, guided by the account of Tawara, was he able to confirm its presence [5]. Our own dissections, as shown in Figure 10, have been validated by histological sectioning, but it remains difficult to be sure of the precise relationships of the different components relative to the gross anatomical landmarks. The anatomical landmarks themselves are also less than well appreciated. The value of the tendon of Todaro and the septal leaflet of the tricuspid valve as forming the boundaries of the nodal triangle, nonetheless, were emphasised by Koch [46] shortly after the initial description provided by Tawara [6]. The fact that the right atrial wall of the triangle then formed one of the boundaries of the inferior pyramidal space was then emphasised by Sealy and colleagues [47], although they considered the space to be septal rather than paraseptal. The precise arrangement had been clarified at the turn of the twenty-first century [48]. The relationship of the apex of the pyramidal space to the inferior extension of the infero-septal recess, however, had been less well recognised [31,34]. It is towards the apex of the pyramidal space that the axis is able to penetrate the insulating tissues of the atrioventricular junctions (Figure 12). 

The area of fibrous tissue penetrated is seemingly the atrioventricular component of the membranous septum. In reality, the insulation is provided by a tongue of fibrous tissue which itself produces fibrous continuity between the leaflets of the mitral and tricuspid valves. As was recognised by Keith and Flack [5], this fibrous tissue, along with the membranous septum itself, is derived from the atrioventricular cushions that separate the embryonic atrioventricular canal into the tricuspid and mitral valvar orifices. The overall area of fibrous tissue is usually described nowadays as the central fibrous body. The larger part, aside from the membranous septum, is then said to be the “right fibrous trigone”. Such an approach, however, fails to account for the variations in the make-up of the aortic root and its relations with the mitral valve. 

**Figure 13:**
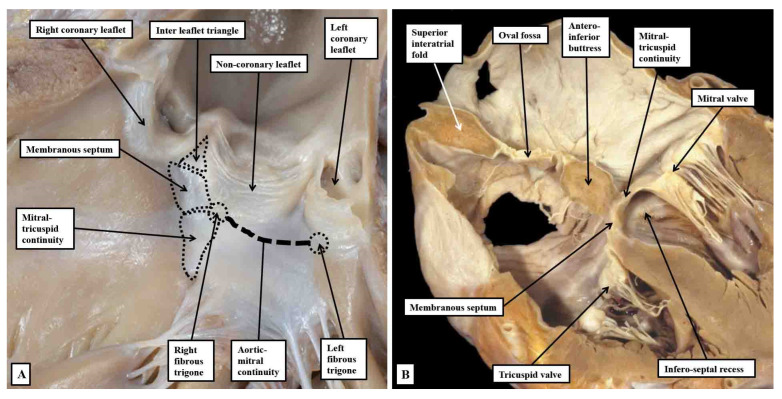
Panel (**A**) shows a view of the opened left ventricular outflow tract of an infant heart viewed from the front. The parts have been marked to show how the so-called central fibrous body is made up of the membranous septum, the area of mitral-to-tricuspid continuity that forms the roof of the infero-septal recess, and the rightward end of the area of fibrous continuity between the leaflets of the aortic and mitral valves, with that component defined as the right fibrous trigone. The leftward end is the left fibrous trigone. The central fibrous body is also directly continuous with the interleaflet triangle between the right and non-coronary sinuses of the aortic root. Panel (**B**) is a “four chamber” section of an adult heart showing how the roof of the infero-septal recess supports the antero-inferior buttress of the atrial septum.

In the majority of hearts, there is fibrous continuity between the leaflets of the aortic and mitral valves. It is this continuity that permits the leaflets of the mitral valve itself to be distinguished as being aortic and mural. It is then the thickenings at the end of this region of fibrous continuity that can be described as the right and left fibrous trigones (Figure 13A). The left fibrous trigone anchors the leftward end of the fibrous continuity to the parietal wall of the left ventricle. The thickening at the rightward end of the area of continuity, in contrast, becomes continuous with the area of continuity between the leaflets of the mitral and tricuspid valves that, when viewed from the cavity of the left ventricle, forms the roof of the infero-septal septal recess (Figure 13B).

**Figure 14:**
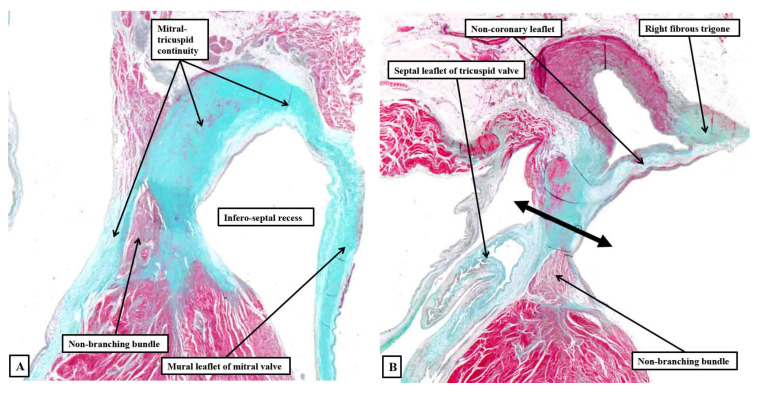
Serial histological sections taken through the infero-septal recess of an adult heart, orientated to show the long axis of the aortic root, with the cavities of the right-sided chambers seen to the left hand. Panel (**A**) shows how the area of mitral-to-tricuspid continuity forms the roof of the recess, with the membranous septum forming its rightward wall, the non-branching bundle occupying the septal component in this section. Panel (**B**) is an anterior and cephalad section cutting through the base of the non-coronary aortic sinus. It shows how the rightward end of the area of fibrous continuity between the aortic and mitral valvar leaflets forms the right fibrous trigone. The double-headed arrow shows the atrioventricular component of the membranous septum forming the rightward wall of the recess, with the non-branching bundle now occupying the interventricular component of the membranous septum.

It is this extensive area of continuity, which also provides the basal support for the antero-inferior buttress of the atrial septum, which makes up the greater part of the central fibrous body. Taken overall, therefore, the central fibrous body is best considered as having parts made up of the right fibrous trigone, the membranous septum, and the area of tricuspid-to-mitral fibrous continuity (Figure 14). 

The tissue that insulates the axis as it transitions from the node to become the non-branching bundle is part of this area of tricuspid-to-mitral fibrous continuity (Figure 14A). There is, furthermore, marked variability in the fashion in which these components contribute to the central fibrous body. Together with other variables, such as the extent of rotation of the aortic root, it is these features that, when properly analysed, are able provide a guide to the potential vulnerability of the conduction axis during transcatheter replacement of the aortic valve [35,49]. 

## 6. Putting the Conduction Axis back into the Heart 

As we have shown, it is possible, using serial histological sections, to identify with precision the components of the atrioventricular conduction axis. It is also possible, when assessing the sections, to identify the gross anatomical landmarks of the areas containing and enclosing these components. As we have already emphasised, however, it is difficult to correlate the location of the axis with precision once the heart has been sectioned histologically. We have shown that, when using gross dissection, it is feasible to show the general overall arrangement. We have also emphasised, nonetheless, that as skilled an anatomist as Keith was unable, in his first attempts, to identify the non-branching bundle when using the technique of gross anatomical dissection [4]. Even when using histological sections, it is difficult to place the conduction axis with precision so as to locate its location both to the landmarks of the right atrium and the aortic root [34]. When using a technique that did permit a more accurate placement of the conduction axis within the heart, furthermore, we demonstrated marked variation of the axis relative to the aortic root depending on the rotation of the root itself within the base of the ventricular mass, the extent of the infero-septal recess, and the degree of outflow tract myocardium incorporated into the left ventricular outflow tract [35]. It would be very helpful if a technique was available that permitted the reconstruction of the heart being analysed, and at the same time permitted the recognition of the precise location of the conduction axis. The technique of microcomputed tomography satisfies these requirements, but also requires the heart being analysed to be immersed in a contrast solution so as to distinguish the conduction axis from the adjacent working myocardium [50]. The alternative techniques of multiscale and hierarchical phase contrast tomography, abbreviated to HiP-CT [51,52], will avoid the need to immerse the specimen under investigation in potentially damaging solutions. The use of these new forms of anatomic imaging, furthermore, will make it possible to reconstruct the heart and then section it in any required plane and at a high resolution, allowing the cellular make-up of the components of the conduction axis to be shown in the three orthogonal planes and revealing the location of the axis with absolute precision relative to the boundaries of the pyramid of Koch. They promise to have the ability to provide key information regarding the relationship of the fascicles of the left bundle branches relative to the components of the aortic root. Positioning the axis within the reconstructed heart will hopefully show the location of the conducting components as seen from either the right or the left sides. In this way, these new techniques, ideally, will provide all the information that is required by those undertaking specific pacing of the axis, as well as those inserting aortic valves using the transcatheter technique. As more hearts are interrogated, it should prove possible to resolve additional problems, such as the precise dimensions of the potential pathways for atrioventricular nodal re-entry tachycardia. As already suggested by those describing the initial results [52], the HiP-CT technique truly has the capacity to be the game-changer required to show the precise arrangement, locations, and variability of the atrioventricular conduction axis. 

## Data Availability

Not Applicable.
